# Supplementary material for: The Identification and Role of the Key Mycotoxin of Pestalotiopsis kenyana Causing Leaf Spot Disease of Zanthoxylum schinifolium
Source: J Fungi (Basel). 2023 Dec 13;9(12):1194. doi: 10.3390/jof9121194 (PMC10744368; doi:10.3390/jof9121194)
Supplement: Supplementary file 1 [file jof-09-01194-s001.zip › Figure S3. The incidence of PK-3 mycotoxin application of Z. schinifolium 5d.pdf]

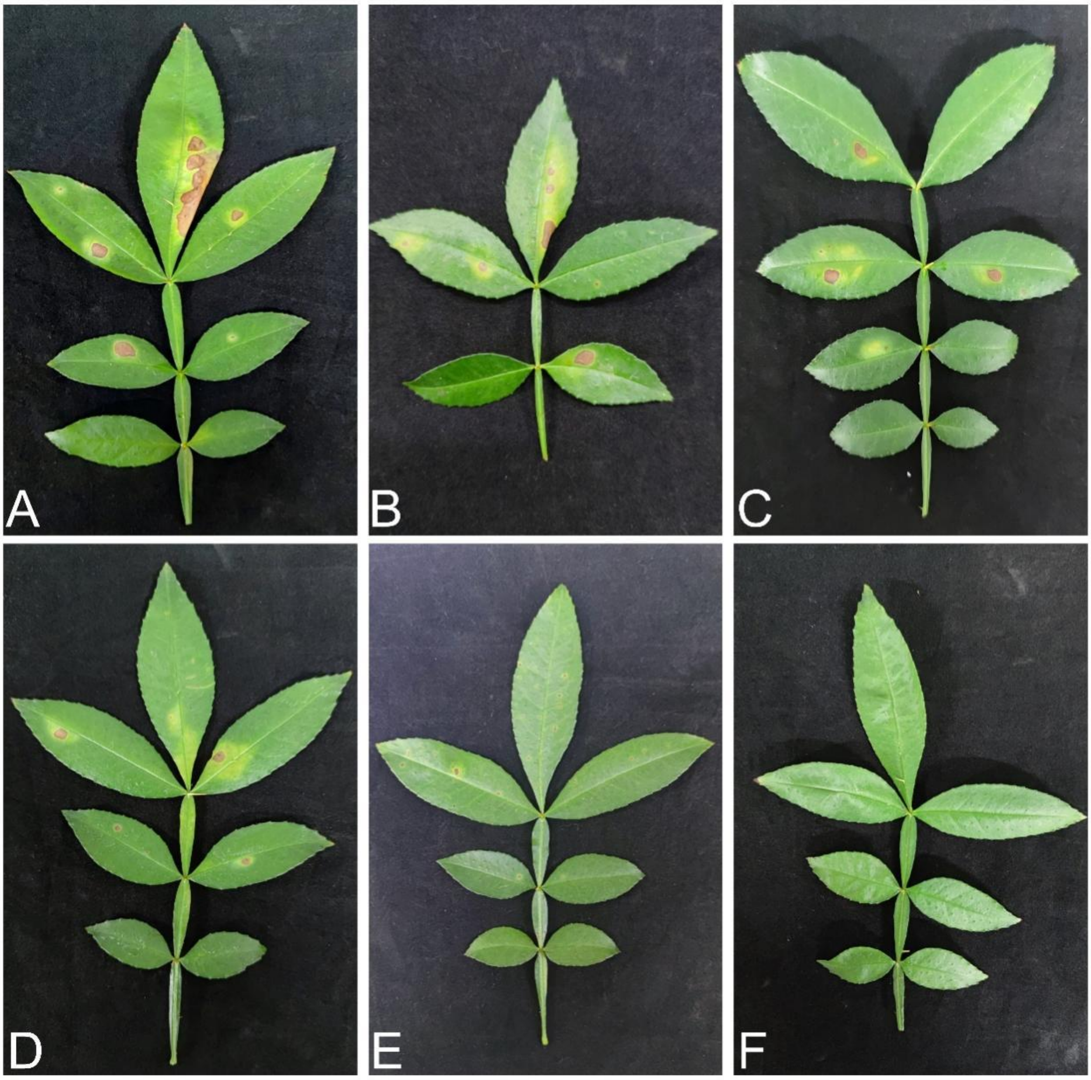

Figure S3. The incidence of PK-3 mycotoxin application of *Z. schinifolium* 5d

A, 10 μg/mL mycotoxin; B, 20 μg/mL mycotoxin; C, 40 μg/mL mycotoxin; D, 80 μg/mL mycotoxin; E, 1 × 10^6 cfu / ml pathogen spore suspension; F, sterile water
