# Supplementary material for: The Identification and Role of the Key Mycotoxin of Pestalotiopsis kenyana Causing Leaf Spot Disease of Zanthoxylum schinifolium
Source: J Fungi (Basel). 2023 Dec 13;9(12):1194. doi: 10.3390/jof9121194 (PMC10744368; doi:10.3390/jof9121194)
Supplement: Supplementary file 1 [file jof-09-01194-s001.zip › Table S1. The ratio of developing agents.pdf]

Table S1. The ratio of developing agents

Solvent	proportion
Petroleum ether: ethyl acetate	2:1; 4:1; 6:1; 8:1; 10:1
Petroleum ether: isoamyl alcohol	2:1; 4:1; 6:1; 8:1; 10:1
Petroleum ether: ethyl acetate: dichloromethane	1:2:1; 1:4:1; 1:6:1; 1:8:1; 1:10:1
Ethyl acetate: ice acetic acid: dichloromethane	2:1:1; 4:1:1; 6:1:1; 8:1:1; 10:1:1
Ethyl acetate: glacial acetic acid: cyclohexane	2:1:1; 4:1:1; 6:1:1; 8:1:1; 10:1:1
Ethyl acetate: glacial acetic acid: hexane	2:1:1; 4:1:1; 6:1:1; 8:1:1; 10:1:1
n-Butanol: glacial acetic acid: dichloromethane	2:1:1; 4:1:1; 6:1:1; 8:1:1; 10:1:1
n-Butanol: glacial acetic acid: petroleum ether	2:1:1; 4:1:1; 6:1:1; 8:1:1; 10:1:1
n-Butanol: glacial acetic acid	2:1; 4:1; 6:1; 8:1; 10:1
