# Supplementary material for: The Identification and Role of the Key Mycotoxin of Pestalotiopsis kenyana Causing Leaf Spot Disease of Zanthoxylum schinifolium
Source: J Fungi (Basel). 2023 Dec 13;9(12):1194. doi: 10.3390/jof9121194 (PMC10744368; doi:10.3390/jof9121194)
Supplement: Supplementary file 1 [file jof-09-01194-s001.zip › Table S2. Ammonium sulfate fractional precipitation toxicity test results.pdf]

Table S2. Ammonium sulfate fractional precipitation toxicity test results.

Saturation (%)	Fraction	Time(h)		
		48	96	144
20	Supernatant	+	+	+
	Precipitation	-	-	-
30	Supernatant	+	+	+
	Precipitation	-	-	-
40	Supernatant	+	+	+
	Precipitation	-	-	-
50	Supernatant	+	+	+
	Precipitation	-	-	-
60	Supernatant	+	+	+
	Precipitation	-	-	-
70	Supernatant	+	+	+
	Precipitation	-	-	-
80	Supernatant	+	+	+
	Precipitation	-	-	-

Note: Saturation: Saturation of ammonium sulfate; ' - ' in the table indicates no obvious symptoms; ' + ' means that the leaves have lesion.
