# Supplementary figures and images for: The Identification and Role of the Key Mycotoxin of Pestalotiopsis kenyana Causing Leaf Spot Disease of Zanthoxylum schinifolium
Source: J Fungi (Basel). 2023 Dec 13;9(12):1194. doi: 10.3390/jof9121194 (PMC10744368; doi:10.3390/jof9121194)

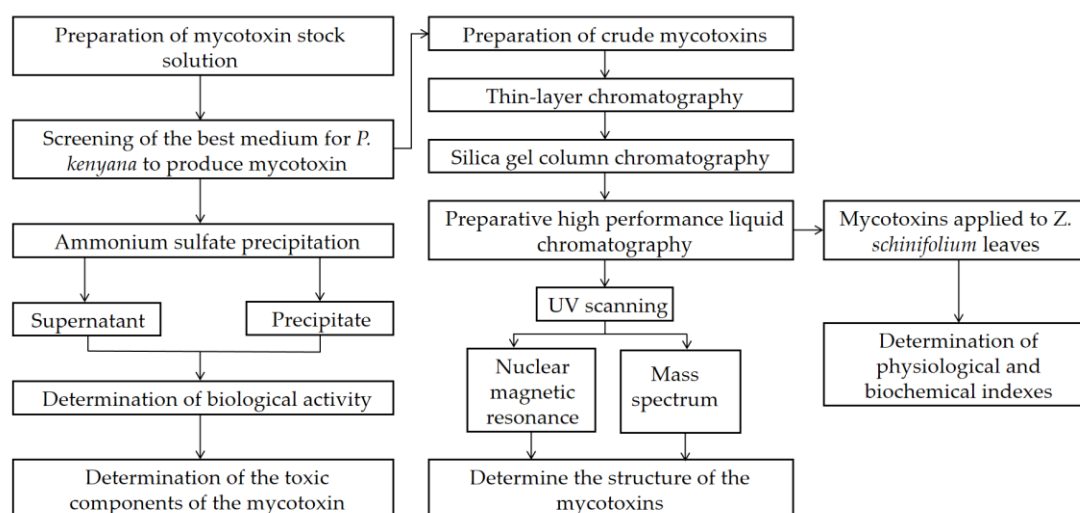

**Figure S1.** Flow figure of the experiment

Supplement: Supplementary file 1 [file jof-09-01194-s001.zip › Figure S1. Flow figure of the experiment.pdf]

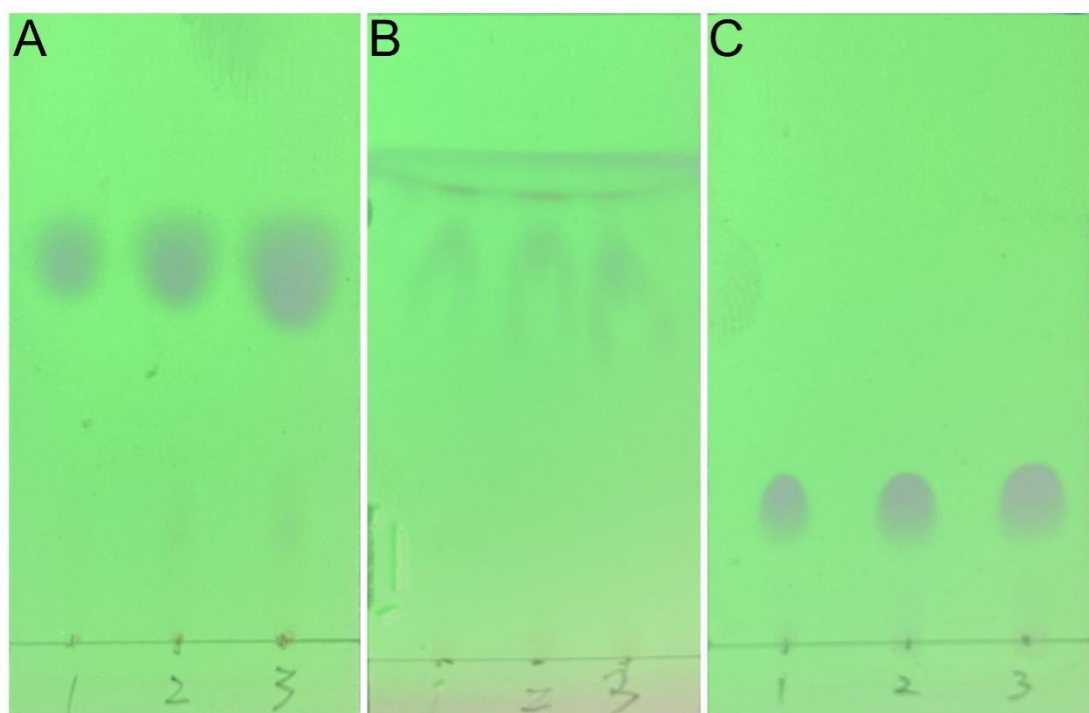

**Figure S2.** Thin layer chromatography detection diagram

A, PK-1; B, PK-2; C, PK-3

Supplement: Supplementary file 1 [file jof-09-01194-s001.zip › Figure S2. Thin layer chromatography detection diagram.pdf]
